# Genomic variants link to hepatitis C racial disparities

**DOI:** 10.18632/oncotarget.19755

**Published:** 2017-08-01

**Authors:** Matthew M. Yeh, Sarag Boukhar, Benjamin Roberts, Nairanjana Dasgupta, Sayed S. Daoud

**Affiliations:** ^1^ Department of Pathology, University of Washington School of Medicine, Seattle, WA 98195, USA; ^2^ The Liver Center, University of Kansas Medical Center, Kansas City, KS 66160, USA; ^3^ Department of Mathematics and Statistics, Washington State University, Pullman, WA 99164, USA; ^4^ Department of Pharmaceutical Sciences, Washington State University Health Sciences, Spokane, WA 99210, USA

**Keywords:** hepatitis C, racial disparity, genomic variants, hepatocellular carcinoma, alternative splicing

## Abstract

Chronic liver diseases are one of the major public health issues in United States, and there are substantial racial disparities in liver cancer-related mortality. We previously identified racially distinct alterations in the expression of transcripts and proteins of hepatitis C (HCV)-induced hepatocellular carcinoma (HCC) between Caucasian (CA) and African American (AA) subgroups. Here, we performed a comparative genome-wide analysis of normal *vs*. HCV+ (cirrhotic state), and normal adjacent tissues (HCCN) *vs*. HCV+HCC (tumor state) of CA at the gene and alternative splicing levels using Affymetrix Human Transcriptome Array (HTA2.0). Many genes and splice variants were abnormally expressed in HCV+ more than in HCV+HCC state compared with normal tissues. Known biological pathways related to cell cycle regulations were altered in HCV+HCC, whereas acute phase reactants were deregulated in HCV+ state. We confirmed by quantitative RT-PCR that *SAA1*, *PCNA-AS1*, *DAB2*, and *IFI30* are differentially deregulated, especially in AA compared with CA samples. Likewise, IHC staining analysis revealed altered expression patterns of SAA1 and HNF4α isoforms in HCV+ liver samples of AA compared with CA. These results demonstrate that several splice variants are primarily deregulated in normal *vs*. HCV+ stage, which is certainly in line with the recent observations showing that the pre-mRNA splicing machinery may be profoundly remodeled during disease progression, and may, therefore, play a major role in HCV racial disparity. The confirmation that certain genes are deregulated in AA compared to CA tissues also suggests that there is a biological basis for the observed racial disparities.

## INTRODUCTION

Hepatocellular carcinoma (HCC) is one of the few malignancies in which the incidence is on the rise worldwide, especially in the US [1]. The increasing incidence of HCC in the US is associated with the rise in Hepatitis C virus (HCV) infection [2]. It is estimated that 3.2 million people in the US are infected with HCV, a blood-borne disease linked to 12,000 US deaths a year [3]. Even with the availability of new oral direct acting antiviral drugs [4], it is anticipated that 320,000 patients will die from HCV, 157,000 will develop HCC, and 203,000 will develop cirrhosis in the next 35 years [5]. Inequalities in disease prevalence, treatment, and outcome make HCC an important health problem among minority groups [6]. First, there are disparities in the prevalence of HCV infection with African Americans (AA) being twice as likely to have been infected compared with Caucasian Americans (CA) [7]. Second, there are significant racial/ethnic disparities in access to HCV care [8]. Third, African Americans are also less likely to respond to the new anti-HCV therapy than Caucasian Americans, possibly due to a lower rate of sustained virologic response (SVR) [9], and have considerably lower likelihood of receiving liver transplantation [10]. While much of the existing literature so far has focused on noting the presence of these disparities, little is known about specific biological or genetic factors that are involved. Therefore, there is clear need for molecular/biological approaches to understand the molecular basis for HCV health and racial disparities. Ultimately positive outcomes would allow for the development of novel, affordable and much needed next generation therapeutic care management based on HCV disease state and the racial/ethnic background of patients [11]. We recently reported that racially distinct alterations in the expression of transcripts and proteins exist between CA and AA individuals infected with HCV, as measured by proteomics-based analysis [12]. For example, we showed that the mRNA levels of transferrin (*TF*), Apolipoprotein A1 (*APOA1*) and hepatocyte nuclear factor 4-alpha (*HNF4α*) were significantly altered in AA liver (cirrhotic) and tumor samples compared to CA. It is known that AA with chronic HCV commonly have elevated levels of serum markers of iron stores and altered cholesterol & triglyceride levels [13, 14]. The expression of *TF* & *APOA1* (both involved in iron homeostasis and lipid metabolic processes, respectively) is transcriptionally regulated by *HNF4α* [15, 16]. Furthermore, *HNF4α* is also known to be involved in the pathogenesis of HCC [17, 18]. To the best of our knowledge, that was the first study to demonstrate possible link between deregulation of the expression of specific transcripts & proteins and HCV racial disparity between AA and CA subgroups. This finding prompted us to further investigate whether alternative splicing (AS) of genes could be involved in the transcriptome diversity seen between these two ethnic populations. Alternative splicing (AS) is a post-transcriptional event whereby exons are joined by different combinations generating various isoforms from a single gene [19–21]. It has been shown that most genes have at least 2 alternative isoforms [22, 23] contributing to both transcriptome and proteome diversities in various pathophysiological situations including HCV infection and HCC [24, 25].

In this study, we have performed a genome-wide transcriptomic analysis at the gene and splice variants levels in liver and tumor tissue samples of HCV infected individuals using the Affymetrix GeneChip Human Transcriptome array (HTA2.0). The array is especially designed to allow for expression profiling of transcript splice variants. It contains >6.0 million probes covering coding transcripts (70%) and exon-exon splice junctions and non-coding transcripts (30%). Herein, we describe our methods for expression microarray analysis at the genes and splice variants levels using Transcriptome Analysis Console (TAC2.0) software coupled by validation studies to confirm disease-specific splice variants of genes that could be involved in the racial disparity of HCV-induced HCC by real-time qRT-PCR and immunohistochemistry using sixty liver and tumor tissue samples.

## RESULTS

### Clinical characteristics of tissue samples

A total of 36 snapped frozen liver and tumor samples from CA and AA populations were used in this study. The clinicopathologic characteristics of samples are presented in Supplementary Table 2. As reported in our previous study [12], there were no significant differences of age and sex between samples in the two groups. However, the cirrhotic HCV+ liver samples of AA group had statistically significant laboratory results for aspartate aminotransferase (AST), and alanine aminotransferase (ALT) (*p*<0.05) compared to CA group. There were no significance differences in the laboratory values for albumin, total albumin and hemoglobin between samples in the two groups.

### Identification of differentially expressed genes and splice variants based on diseased states of Caucasian American (CA) population

**Figure 1:**
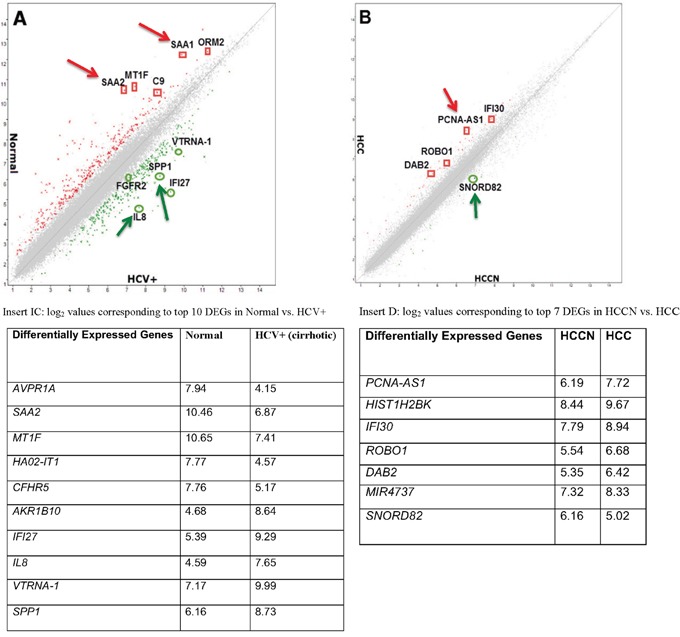
Global gene expression profiling data of hepatitis C tissue samples **(A)**: Scatter plot presenting the values of *log_2_* for each gene in the normal (Y-axis) *vs*. HCV+ cirrhotic samples (X-axis). **(B)**: Scatter plot presenting the values of *log_2_* for each gene in the HCCN (X-axis) *vs*. HCV+HCC tumor samples (Y-axis). Insert **(C)**: Table indicating the *log2* values corresponding to top 10 DEGs in normal *vs*. HCV+ samples. Insert **(D)**: Table indicating the *log2* values corresponding to top 7 DEGs in HCCN *vs*. HCV+ HCC samples.

Gene level differential expression profiles of 12 CA tissues samples (3 normal liver, 3 HCV+ livers, 3 HCV+/HCC+ tumors and 3 HCCN) were determined using HTA2.0 GeneChip Arrays (Affymetrix®) that contain 70,523 detectable transcripts using TAC2.0 software (for filtering criteria see Materials and methods). For normal *vs*. HCV+, 636 genes were differentially expressed: 350 genes were up-regulated in HCV+ compared to normal (coding 235; non-coding 103; other 12) as shown in Table 1A, whereas 286 genes were down-regulated in HCV+ compared to normal (coding 209; non-coding 73; other 4), Table 1B. For HCCN *vs*. HCV+HCC, only 61 genes were differentially expressed, as shown in Table 2, using the same algorithm options and filter criteria (see Materials and methods): 47 genes were up-regulated in HCV+HCC compared to HCCN (coding 23; non-coding 6; other 18) and 14 genes were down-regulated in HCV+HCC compared to HCCN (coding 5; non-coding 1; other 8). These results suggest that tumor-adjacent tissue (HCCN) shares biology of the tumors themselves, and only 61 genes are differentially expressed in this case. Figure 1 shows the scatter plot (*log* 2 scale of expression values) for differentially expressed genes (DEGs) in normal *vs*. HCV+ state (Figure 1A) and HCCN *vs*. HCV+HCC state (Figure 1B), respectively. In both cases, most of the genes run along the diagonal axis and can be considered as common genes, expressed similarly in either diseased state, whereas differentially expressed genes with values <-2.0 or <+2.0 are scattered outside the diagonal axis. Examples of these scattered genes (arrows) are shown in Figure 1A (insert 1 C) and Figure 1B (insert 1 D). No overlap of genes (marked) was detected between the two disease stages, which suggest that these genes are differentially expressed based on disease state (normal *vs*. HCV+ cirrhotic livers; HCCN *vs*. HCV+/HCC cirrhotic tumors).

**Table 1A:** The results of differentially expressed genes (DEGs) in normal *vs*. HCV+ tissue samples

Accession Number	Fold Change	Fold Direction	*p* value	Gene Symbol	Group
NM_000706	13.8	N UP vs. HCV	0.01640	AVPR1A	Coding
NM_030754	12.05	N UP vs. HCV	0.00282	SAA2	Coding
NM_005949	9.48	N UP vs. HCV	0.04235	MT1F	Coding
NM_030787	6.01	N UP vs. HCV	0.00645	CFHR5	Coding
NM_014926	5.96	N UP vs. HCV	0.01872	SLITRK3	Coding
NM_001144904	5.79	N UP vs. HCV	0.03399	CLEC4M	Coding
NM_000331	5.13	N UP vs. HCV	0.01927	SAA1	Coding
NM_001166624	5.08	N UP vs. HCV	0.01142	CFHR3	Coding
NM_001201550	4.99	N UP vs. HCV	0.02009	CFHR4	Coding
NM_176870	4.45	N UP vs. HCV	0.02094	MT1M	Coding
NM_001308	3.97	N UP vs. HCV	0.03343	CPN1	Coding
NM_001146726	3.93	N UP vs. HCV	0.00794	TIMD4	Coding
NM_145290	3.68	N UP vs. HCV	0.00611	GPR125	Coding
NM_031900	3.62	N UP vs. HCV	0.01828	AGXT2	Coding
NM_020459	3.54	N UP vs. HCV	0.02778	PAIP2B	Coding
NM_032649	3.52	N UP vs. HCV	0.00289	CNDP1	Coding
NM_001159	3.45	N UP vs. HCV	0.02937	AOX1	Coding
NM_001361	3.31	N UP vs. HCV	0.01586	DHODH	Coding
NM_006419	3.3	N UP vs. HCV	0.00101	CXCL13	Coding
NM_001039199	3.29	N UP vs. HCV	0.00756	TTPAL	Coding
NM_001127708	3.29	N UP vs. HCV	0.03135	PRG4	Coding
NM_001193646	3.28	N UP vs. HCV	0.04037	ATF5	Coding
NM_001143838	3.27	N UP vs. HCV	0.04855	SLC13A5	Coding
NM_052972	3.25	N UP vs. HCV	0.00249	LRG1	Coding
NM_000028	3.2	N UP vs. HCV	0.00334	AGL	Coding
NM_000055	3.11	N UP vs. HCV	0.01262	BCHE	Coding
NM_175737	3.09	N UP vs. HCV	0.02281	KLB	Coding
NM_000902	2.99	N UP vs. HCV	0.00453	MME	Coding
NM_016371	2.97	N UP vs. HCV	0.04476	HSD17B7	Coding
NM_018078	2.95	N UP vs. HCV	0.04017	LARP1B	Coding
NM_000133	2.93	N UP vs. HCV	0.04671	F9	Coding
NM_001170701	2.9	N UP vs. HCV	0.00523	MBLN3	Coding
NM_004944	2.89	N UP vs. HCV	0.03243	DNASE1L3	Coding
NM_006691	2.81	N UP vs. HCV	0.00779	LYVE1	Coding
NM_014465	2.79	N UP vs. HCV	0.00251	SULT1B1	Coding
NM_001161429	2.7	N UP vs. HCV	0.00854	RANBP3L	Coding
NM_006770	2.69	N UP vs. HCV	0.01995	MARCO	Coding
NM_001174152	2.68	N UP vs. HCV	0.00824	RABEPK	Coding
NM_001130991	2.62	N UP vs. HCV	0.00355	HYOU1	Coding
NM_033058	2.59	N UP vs. HCV	0.04228	TRIM55	Coding
NM_001123	2.54	N UP vs. HCV	0.02600	ADK	Coding
NM_004169	2.52	N UP vs. HCV	0.00361	SHMT1	Coding
NM_005907	2.5	N UP vs. HCV	0.00967	MAN1A1	Coding
NM_001128431	2.5	N UP vs. HCV	0.01099	SLC39A14	Coding
NM_001128227	2.5	N UP vs. HCV	0.01359	GNE	Coding
NM_001737	2.49	N UP vs. HCV	0.01724	C9	Coding
NM_004911	2.47	N UP vs. HCV	0.00481	PDIA4	Coding
NM_000019	2.47	N UP vs. HCV	0.00874	ACAT1	Coding
NM_005768	2.47	N UP vs. HCV	0.03440	LPCAT3	Coding
NM_000066	2.47	N UP vs. HCV	0.04159	C8B	Coding
NM_000478	2.46	N UP vs. HCV	0.00447	ALPL	Coding
NM_145715	2.44	N UP vs. HCV	0.01064	TIGD2	Coding
NM_004481	2.43	N UP vs. HCV	0.03059	GALNT2	Coding
NM_000236	2.43	N UP vs. HCV	0.03763	LIPC	Coding
NM_004475	2.39	N UP vs. HCV	0.00135	FLOT2	Coding
NM_014730	2.38	N UP vs. HCV	0.00073	MLEC	Coding
NM_138326	2.38	N UP vs. HCV	0.03850	ACMSD	Coding
NM_015541	2.37	N UP vs. HCV	0.04555	LRIG1	Coding
NM_003658	2.36	N UP vs. HCV	0.02789	MT1DP	Coding
NM_004108	2.34	N UP vs. HCV	0.01438	FCN2	Coding
NM_001242332	2.32	N UP vs. HCV	0.00197	USP17L6P	Coding
NM_000715	2.32	N UP vs. HCV	0.02707	C4BPA	Coding
NM_001199758	2.31	N UP vs. HCV	0.00640	MTHF5	Coding
NM_001144978	2.31	N UP vs. HCV	0.00910	MTHFD2L	Coding
NM_181536	2.31	N UP vs. HCV	0.02866	PKD1L3	Coding
NM_004388	2.3	N UP vs. HCV	0.00628	CTBS	Coding
NM_005570	2.3	N UP vs. HCV	0.01109	LMAN1	Coding
NM_002168	2.29	N UP vs. HCV	0.00779	IDH2	Coding
NM_000348	2.27	N UP vs. HCV	0.01335	SRD5A2	Coding
NM_000240	2.27	N UP vs. HCV	0.02094	MAO2	Coding
NM_001859	2.27	N UP vs. HCV	0.03664	SLC31A1	Coding
NM_005691	2.26	N UP vs. HCV	0.00742	ABCC9	Coding
NM_001005375	2.26	N UP vs. HCV	0.03061	DAZ4	Coding
NM_000562	2.25	N UP vs. HCV	0.04361	C8A	Coding
NM_000065	2.23	N UP vs. HCV	0.04204	C6	Coding
NM_000608	2.22	N UP vs. HCV	0.01256	ORM2	Coding
NM_039654	2.22	N UP vs. HCV	0.02000	MIR4450	Coding
NM_005794	2.21	N UP vs. HCV	0.00033	DHRS2	Coding
NM_022132	2.19	N UP vs. HCV	0.01297	MCCC2	Coding
NM_030782	2.18	N UP vs. HCV	0.00912	CLPTM1L	Coding
NM_182758	2.18	N UP vs. HCV	0.01132	WDR72	Coding
NM_001014797	2.16	N UP vs. HCV	0.00922	KCNMA1	Coding
NM_006741	2.16	N UP vs. HCV	0.01382	PPP1R1A	Coding
NM_181900	2.16	N UP vs. HCV	0.03056	STARD5	Coding
NM_005013	2.14	N UP vs. HCV	0.02120	NUCB2	Coding
NM_001918	2.13	N UP vs. HCV	0.03126	DBT	Coding
NM_001161504	2.11	N UP vs. HCV	0.02578	ALDH4A1	Coding
NM_001015880	2.1	N UP vs. HCV	0.00207	PAPSS2	Coding
NM_001100607	2.1	N UP vs. HCV	0.01792	SERPINA10	Coding
NM_001145368	2.08	N UP vs. HCV	0.00871	PTPN3	Coding
NM_005045	2.07	N UP vs. HCV	0.00942	RELN	Coding
NM_138493	2.06	N UP vs. HCV	0.00822	CCDC167	Coding
NR_029524	2.06	N UP vs. HCV	0.01216	MIR107	Coding
NM_001113239	2.02	N UP vs. HCV	0.00036	HIPK2	Coding
NM_003878	2.02	N UP vs. HCV	0.00058	GGH	Coding
NM_001872	2.01	N UP vs. HCV	0.04171	CPB2	Coding
NM_021800	2.01	N UP vs. HCV	0.04931	DNAJC12	Coding

**Table 1B:** The results of differentially expressed genes (DEGs) in HCV+ *vs*. Normal tissue samples

Accession Number	Fold Change	Fold Direction	*p* value	Gene Symbol	Group
NM_020299	-30.81	HCV UP vs. N	0.00242	AKR1B10	Coding
NM_001130080	-14.86	HCV UP vs. N	0.02019	IFI27	Coding
NM_000584	-8.33	HCV UP vs. N	0.03313	IL8	Coding
NR_026703	-7.05	HCV UP vs. N	0.02314	VTRNA1-1	Coding
NM_000582	-6.02	HCV UP vs. N	0.03381	SPP1	Coding
NM_004864	-5.65	HCV UP vs. N	0.00097	GDF15	Coding
NM_033049	-5.46	HCV UP vs. N	0.03079	MUC13	Coding
NM_001040092	-4.93	HCV UP vs. N	0.00379	ENPP2	Coding
NM_001565	-4.79	HCV UP vs. N	0.00803	CXCL10	Coding
NM_006149	-3.89	HCV UP vs. N	0.00061	LGALS4	Coding
NM_001046	-3.84	HCV UP vs. N	0.02276	SLC12A2	Coding
NR_002921	-3.83	HCV UP vs. N	0.00306	SNORA75	Coding
NM_006398	-3.77	HCV UP vs. N	0.04837	UBD	Coding
NM_025130	-3.66	HCV UP vs. N	0.02106	HKDC1	Coding
NM_000492	-3.61	HCV UP vs. N	0.00914	CFTR	Coding
NM_000552	-3.59	HCV UP vs. N	0.00285	VWF	Coding
NR_002953	-3.45	HCV UP vs. N	0.00506	SNORA11	Coding
NM_001128175	-3.39	HCV UP vs. N	0.00364	DTNA	Coding
NM_031310	-3.38	HCV UP vs. N	0.00235	PLVAP	Coding
AF533910	-3.33	HCV UP vs. N	0.04893	HLA-DQA1	Coding
NR_002915	-3.3	HCV UP vs. N	0.00041	SNORA74A	Coding
NM_001166395	-3.29	HCV UP vs. N	0.00387	CHST4	Coding
AF287958	-3.29	HCV UP vs. N	0.01057	HLA-A	Coding
NM_016591	-3.26	HCV UP vs. N	0.03060	BICC1	Coding
NM_005245	-3.21	HCV UP vs. N	0.01618	FAT1	Coding
NM_144975	-3.2	HCV UP vs. N	0.01512	SLFN5	Coding
NM_021983	-3.11	HCV UP vs. N	0.01176	HLA-DRB4	Coding
NR_003016	-3.09	HCV UP vs. N	0.02789	SNORA26	Coding
NM_005567	-3.05	HCV UP vs. N	0.00582	LGALS3BP	Coding
NM_020638	-3.03	HCV UP vs. N	0.02594	FGF23	Coding
NM_006274	-2.95	HCV UP vs. N	0.00198	CCL19	Coding
NM_001901	-2.87	HCV UP vs. N	0.04083	CTGF	Coding
NM_001144964	-2.84	HCV UP vs. N	0.00177	NEDD4L	Coding
NM_001003954	-2.81	HCV UP vs. N	0.00160	ANXA13	Coding
NM_017533	-2.81	HCV UP vs. N	0.02032	MYH4	Coding
NM_005961	-2.73	HCV UP vs. N	0.00874	MUC6	Coding
NM_002345	-2.72	HCV UP vs. N	0.02683	LUM	Coding
NM_001164617	-2.71	HCV UP vs. N	0.03061	GPC3	Coding
NM_138694	-2.68	HCV UP vs. N	0.00081	PKHD1	Coding
NM_001206567	-2.68	HCV UP vs. N	0.00272	IFI16	Coding
NM_001242758	-2.68	HCV UP vs. N	0.00823	HLA-A	Coding
NM_002354	-2.68	HCV UP vs. N	0.02366	EPCAM	Coding
NM_005218	-2.59	HCV UP vs. N	0.03577	DEFB1	Coding
NM_001781	-2.58	HCV UP vs. N	0.03613	CD69	Coding
NM_016548	-2.57	HCV UP vs. N	0.00153	GOLM1	Coding
NM_000587	-2.52	HCV UP vs. N	0.01468	C7	Coding
NM_002867	-2.47	HCV UP vs. N	0.03684	RAB3B	Coding
NM_001546	-2.46	HCV UP vs. N	0.00355	ID4	Coding
NM_005233	-2.45	HCV UP vs. N	0.01517	EPHA3	Coding
NM_005261	-2.43	HCV UP vs. N	0.01036	GEM	Coding
NM_002989	-2.42	HCV UP vs. N	0.00164	CCL21	Coding
NM_002416	-2.37	HCV UP vs. N	0.02732	CXCL9	Coding
NM_005556	-2.37	HCV UP vs. N	0.02828	KRT7	Coding
NM_138788	-2.34	HCV UP vs. N	0.00009	TMEM45B	Coding
NM_015529	-2.34	HCV UP vs. N	0.03311	MOXD1	Coding
NM_032211	-2.28	HCV UP vs. N	0.00438	LOXL4	Coding
NM_000346	-2.28	HCV UP vs. N	0.00737	SOX9	Coding
NM_173648	-2.25	HCV UP vs. N	0.00153	CCDC141	Coding
NM_003319	-2.25	HCV UP vs. N	0.00285	TTN	Coding
NM_003246	-2.23	HCV UP vs. N	0.03008	THBS1	Coding
NM_000366	-2.23	HCV UP vs. N	0.04147	TPM1	Coding
NM_001198695	-2.17	HCV UP vs. N	0.00717	MFAP4	Coding
NM_001128310	-2.17	HCV UP vs. N	0.01904	SPARCL1	Coding
NM_001105549	-2.16	HCV UP vs. N	0.00629	ZNF83	Coding
NM_003897	-2.15	HCV UP vs. N	0.01088	IER3	Coding
NM_004791	-2.15	HCV UP vs. N	0.04359	ITGBL1	Coding
NM_001005180	-2.14	HCV UP vs. N	0.00085	TRIM22	Coding
NM_018420	-2.14	HCV UP vs. N	0.01240	SLC22A15	Coding
NM_005841	-2.14	HCV UP vs. N	0.01787	SPRY1	Coding
NM_182832	-2.14	HCV UP vs. N	0.04488	PLAC4	Coding
NM_002392	-2.13	HCV UP vs. N	0.00520	MDM2	Coding
NM_001080538	-2.13	HCV UP vs. N	0.01548	AKR1B15	Coding
NM_014314	-2.13	HCV UP vs. N	0.02827	DDX58	Coding
NM_000141	-2.09	HCV UP vs. N	0.00133	FGFR2	Coding
NM_006291	-2.09	HCV UP vs. N	0.03200	TNFAIP2	Coding
NM_001129	-2.07	HCV UP vs. N	0.04471	AEBP1	Coding
NM_001005473	-2.06	HCV UP vs. N	0.02827	PLCXD3	Coding
NM_014256	-2.06	HCV UP vs. N	0.04406	B3GNT3	Coding
NM_144682	-2.05	HCV UP vs. N	0.00055	SLFN13	Coding
NM_198281	-2.05	HCV UP vs. N	0.01338	GPRIN3	Coding
NM_001098484	-2.02	HCV UP vs. N	0.01968	SLC4A4	Coding
NM_001253835	-2.01	HCV UP vs. N	0.03487	IGFBP7	Coding

**Table 2:** The results of differentially expressed genes (DEGs) in HCC *vs*. HCCN samples

Accession Number	Fold Change	Fold Direction	*p* value	Gene Symbol	Group
NR_028370	3.53	HCC UP vs. HCCN	0.04806	PCNA-AS1	Coding
NM_080593	2.35	HCC UP vs. HCCN	0.04926	HIST1H2BK	Coding
NM_006332	2.21	HCC UP vs. HCCN	0.04400	IFI30	Coding
NM_001145845	2.2	HCC UP vs. HCCN	0.03077	ROBO1	Coding
NM_001244871	2.11	HCC UP vs. HCCN	0.04974	DAB2	Coding
NR_039890	2.01	HCC UP vs. HCCN	0.03285	MIR4737	Coding
NR_004398	-2.20	HCC UP vs. HCCN	0.01972	SNORD82	Coding

For alternative splicing analysis, based on the algorithm options and filter criteria stated in the materials and methods, we were able to detect splice variant events only in normal *vs*. HCV+ stage (cirrhotic) and not in HCCN *vs*. HCV+HCC stage (tumor). This could be due to the low numbers of DEGs detected in the tumor state (61 genes) and/or the cut off and filter criteria. However, in normal *vs*. HCV+ stage about 12,650 genes were expressed in both conditions (coding). Only 15% of genes have at least one PSR or junction with SI (linear) <-2.0 or >+2.0 to indicate alternative splicing. For non-coding, about 2,943 of genes were expressed in both conditions. Only 2.7% of genes were found to have at least one PSR or junction with SI (linear) <-2.0 or >+2.0 to indicate alternative splicing. Table 3 shows various alternative splicing events (coding) for the top 30 genes identified in normal *vs*. HCV+ livers.

**Table 3:** The results of alternative splicing (AS) events in Normal *vs*. HCV+ tissue samples using Affymetrix Human Transcriptomic Array 2.0 (HTA 2.0)

Accession Number	Fold Change (FC)	Gene Symbol	Group	Splicing Index (SI)*	Splicing Events
NM_005950	10.24	MT1G	Coding	-2.14	Cassette Exon
NM_176870	9.94	MT1M	Coding	-2.37	Cassette Exon
NM_005949	7.44	MT1F	Coding	-2.84	
NM_017460	6.68	CYP3A4	Coding	3.18	
NM_017460	6.68	CYP3A4	Coding	2.19	
NM_017460	6.68	CYP3A4	Coding	-2.03	
NM_017460	6.68	CYP3A4	Coding	-2.22	Cassette Exon
NM_017460	6.68	CYP3A4	Coding	-4.27	Alternative 5' Donor Site
NM_017460	6.68	CYP3A4	Coding	-4.36	
NM_030787	6.44	CFHR5	Coding	2.03	
NM_000669	5.58	ADH1C	Coding	2.08	Alternative 5' Donor Site
NM_000669	5.58	ADH1C	Coding	-4.86	Cassette Exon
NM_001881	4.81	CRHBP	Coding	2.15	Alternative 5' Donor Site
NM_001881	4.81	CRHBP	Coding	-4.8	Cassette Exon
NM_019844	4.74	SLCO1B3	Coding	-2.3	Cassette Exon
NM_019844	4.74	SLCO1B3	Coding	-2.31	
NM_019844	4.74	SLCO1B3	Coding	-2.36	Cassette Exon
NM_019844	4.74	SLCO1B3	Coding	-2.46	
NM_019844	4.74	SLCO1B3	Coding	-2.76	Alternative 3' Acceptor Site
NM_019844	4.74	SLCO1B3	Coding	-3.72	Cassette Exon
NM_019844	4.74	SLCO1B3	Coding	-4.19	Cassette Exon
NM_019844	4.74	SLCO1B3	Coding	-4.4	Cassette Exon
NM_019844	4.74	SLCO1B3	Coding	-4.84	
NM_003708	4.49	RDH16	Coding	-3.3	Alternative 5' Donor Site
NM_177550	4.47	SLC13A5	Coding	2.66	
NM_177550	4.47	SLC13A5	Coding	-2.54	
NM_177550	4.47	SLC13A5	Coding	-5.52	Alternative 3' Acceptor Site
NM_003645	4.42	SLC27A2	Coding	-3.63	
NM_001308	4.37	CPN1	Coding	-2.86	Alternative 3' Acceptor Site
NM_006100	4.36	ST3GAL6	Coding	2.41	
NM_006100	4.36	ST3GAL6	Coding	-2.1	Cassette Exon
NM_006100	4.36	ST3GAL6	Coding	-2.19	Cassette Exon
NM_006100	4.36	ST3GAL6	Coding	-2.21	Cassette Exon
NM_006100	4.36	ST3GAL6	Coding	-2.66	Cassette Exon
NM_006100	4.36	ST3GAL6	Coding	-2.8	Cassette Exon
NM_006100	4.36	ST3GAL6	Coding	-3.23	
NM_006100	4.36	ST3GAL6	Coding	-3.57	Alternative 5' Donor Site
NM_006100	4.36	ST3GAL6	Coding	-3.81	Cassette Exon
NM_006100	4.36	ST3GAL6	Coding	-6.23	Alternative 3' Acceptor Site
NM_004944	4.33	DNASE1L3	Coding	-3.74	Intron Retention
NM_004944	4.33	DNASE1L3	Coding	-5.49	Alternative 5' Donor Site
NM_004944	4.33	DNASE1L3	Coding	-6.67	
NM_018388	4.22	MBNL3	Coding	-2.13	
NM_018388	4.22	MBNL3	Coding	-4.34	Cassette Exon
NM_012068	3.8	ATF5	Coding	-2.2	Alternative 5' Donor Site
NM_012068	3.8	ATF5	Coding	-3.13	Cassette Exon
NM_012068	3.8	ATF5	Coding	-3.2	
NM_030754	3.69	SAA2	Coding	22.12	
NM_030754	3.69	SAA2	Coding	12.96	
NM_030754	3.69	SAA2	Coding	10.89	
NM_030754	3.69	SAA2	Coding	8.47	
NM_030754	3.69	SAA2	Coding	8.4	Intron Retention
NM_030754	3.69	SAA2	Coding	6.78	Cassette Exon
NM_030754	3.69	SAA2	Coding	5.96	
NM_030754	3.69	SAA2	Coding	5.25	Cassette Exon
NM_030754	3.69	SAA2	Coding	5.11	Cassette Exon
NM_030754	3.69	SAA2	Coding	5.01	
NM_030754	3.69	SAA2	Coding	3.97	Cassette Exon
NM_030754	3.69	SAA2	Coding	2.52	
NM_030754	3.69	SAA2	Coding	-2.63	Alternative 5' Donor Site
NM_024039	3.65	MIS12	Coding	-2.1	Cassette Exon
NM_024039	3.65	MIS12	Coding	-2.79	
NM_024039	3.65	MIS12	Coding	-3.67	Alternative 5' Donor Site
NM_005952	3.65	MT1X	Coding	-10.33	Alternative 3' Acceptor Site
NM_005952	3.61	MT1X	Coding	-3.98	Cassette Exon
NM_005952	3.6	MT1X	Coding	-4.2	
NM_005952	3.59	MT1X	Coding	-2.23	Cassette Exon
NM_024331	3.59	TTPAL	Coding	-2.96	
NM_001361	3.54	DHODH	Coding	-2.03	
NM_000236	3.54	LIPC	Coding	-4.04	Alternative 5' Donor Site
NM_000236	3.48	LIPC	Coding	-2.27	Cassette Exon
NM_031900	3.48	AGXT2	Coding	-4.04	
NM_052972	3.41	LRG1	Coding	-3.18	Alternative 5' Donor Site
NM_032565	3.39	EBPL	Coding	-2.06	
NM_032565	3.39	EBPL	Coding	-2.11	Cassette Exon
NM_024641	3.39	MANEA	Coding	-2.2	Cassette Exon
NM_020988	3.39	GNAO1	Coding	-2.2	Cassette Exon
NM_020988	3.39	GNAO1	Coding	-2.97	Cassette Exon
NM_020988	3.37	GNAO1	Coding	-3.68	
NM_020988	3.37	GNAO1	Coding	-2.04	Cassette Exon
NM_020988	3.37	GNAO1	Coding	-2.19	
NM_020988	3.37	GNAO1	Coding	-2.46	
NM_000028	3.37	AGL	Coding	-2.74	Cassette Exon
NM_000028	3.36	AGL	Coding	-2.96	
NM_000028	3.36	AGL	Coding	26.12	
NM_000028	3.36	AGL	Coding	12.96	
NM_000028	3.36	AGL	Coding	8.08	Intron Retention
NM_000331	3.36	SAA1	Coding	4.49	
NM_000331	3.27	SAA1	Coding	2.21	
NM_000331	3.27	SAA1	Coding	-2.05	Cassette Exon
NM_000331	3.27	SAA1	Coding	-2.66	Alternative 5' Donor Site
NM_000331	3.27	SAA1	Coding	-3.06	Alternative 3' Acceptor Site
NM_001159	3.27	AOX1	Coding	-3.42	
NM_015506	3.27	MMACHC	Coding	-3.86	Alternative 5' Donor Site

Results were obtained following data normalization using Affymetrix Transcriptome Analysis Console 2.0 (TAC 2.0) software, which determines the Splicing Index (SI) of a gene and *q*-value <0.05 FC as criteria for selection.

*SI = The ratio of the exon intensities in Normal vs. HCV+ livers after normalization to their respective gene intensities in each sample. SI = (0) value indicates that the Probeset Selection Region (PSR) is present at equal levels in both Normal and HCV+ livers. SI = (+) value implies elevated inclusion, and (-) value suggests increased PSR skipping in Normal vs. HCV+ livers.

### Differentially expressed genes are involved in a number of pathways and networks associated with disease state

To gain insights into the molecular pathways involving the identified differentially expressed genes, Ingenuity Pathway Analysis (IPA) of experimental data was performed by Ingenuity software as we previously reported [12]. Using the list of 636 genes involved in normal *vs*. HCV+ (cirrhotic) events and 61 genes involved in HCCN *vs*. HCV+HCC (tumor) events, IPA identified several pathways and function that might be relevant for each disease stage as shown in Tables 4A and 4B, respectively. Top associated network functions for differentially expressed genes in HCV+ cirrhotic state (Table 4A) were: 1) Hepatic fibrosis/hepatic stellate cell activation, 2) Antigen presentation pathway, 3) Graft-versus-host disease signaling, 4) Inhibition of matrix metalloproteases, and 5) T-helper cell differentiation. These data suggest that acute inflammatory phase is involved in HCV+ cirrhotic state as a result of HCV-induced oxidative stress. Genes such as *SAA1*, *SAA2* and *LGALS4* known to be involved in acute inflammatory phase were detected in this disease state (Tables 1A and 1B; Figure 1A). For HCCN *vs*. HCV+HCC (tumor stage), top associated network functions for differentially expressed genes (Table 4B) were: 1) GADD 45 signaling, 2) Cell cycle control of chromosomal replication, 3) Estrogen-mediated S-phase entry, 4) Cell cycle: G2/M DNA damage checkpoint regulation, 5) Cyclins and cell cycle regulation. These data suggest that cell cycle signaling pathways are certainly involved in HCV-induced HCC (tumor phase). Genes such as *PCNA-AS1* and *HIST1H2BK* known to be involved in cell cycle regulation pathways were detected in this disease stage (Table 2; Figure 1B).

**Table 4A:** Functional analysis of 636 differentially expressed genes (DEGs) between Normal vs. HCV+ tissue samples

Top Canonical Pathways		
**Name**	***p*-value**	**ratio**
Hepatic Fibrosis/Hepatic Stellate Cell Activation	4.25E-04	28/127 (0.22)
Antigen Presentation Pathway	4.34E-04	8/18 (0.44)
Graft-versus-Host Disease Signaling	1.48E-03	8/21 (0.381)
Inhibition of Matrix Metalloproteases	2.89E-03	8/23 (0.348)
T Helper Cell Differentiation	3.37E-03	11/39 (0.282)
**Top Toxicity Functions**		
**Name**	***p*-value**	**# Molecules**
Liver Cirrhosis	4.96E-03 – 4.96E-03	5
Liver Necrosis/Cell Death	1.01E-01 – 1.01E-01	4
Liver Adhesion	1.14E-01 – 1.14E-01	1
Liver Fibrosis	2.16E-01 – 6.22E-01	3
Liver Proliferation	2.16E-01 – 6.22E-01	3
**Molecular and Cellular Functions**		
**Name**	***p*-value**	**# Molecules**
DNA Replication, Recombination, and Repair	2.29E-02 – 2.29E-02	3

**Table 4B:** Functional analysis of 61 differentially expressed genes (DEGs) between HCCN vs. HCC tissue samples

Top Canonical Pathways		
**Name**	***p*-value**	**ratio**
GADD45 Signaling	2.93E-06	8/19 (0.421)
Cell Cycle Control of Chromosomal Replication	1.07E-05	8/22 (0.364)
Estrogen-mediated S-Phase Entry	2.24E-05	8/24 (0.333)
Cell Cycle: G2/M DNA Damage Checkpoint Regulation	2.31E-05	11/46 (0.239)
Cyclins and Cell Cycle Regulation	6.44E-05	13/69 (0.188)
**Top Toxicity Functions**		
**Name**	***p*-value**	**# Molecules**
Hepatocellular Carcinoma	3.50E-03 – 5.87E-01	9
Liver Hyperplasia/Hyperproliferation	3.50E-03 – 5.87E-01	31
Glutathione Depletion in Liver	5.37E-02 – 5.38E-01	2
Liver Damage	5.37E-02 – 3.92E-01	7
Liver Degradation	5.37E-02 – 5.37E-02	1
**Molecular and Cellular Functions**		
**Name**	***p*-value**	**# Molecules**
Carbohydrate Metabolism	1.42E-03 – 1.42E-03	3
Drug Metabolism	1.42E-03 – 1.42E-03	3
Molecular Transport	1.42E-03 – 3.73E-02	7
Small Molecule Biochemistry	1.42E-03 – 3.73E-02	10
Post-Translational Modification	2.88E-03 – 2.88E-03	2

Target validation of gene expression and splice variants in Caucasian and African Americans tissue samples

In order to determine whether the racial disparity seen in HCV associated HCC is partly due to the diversity in gene expression and splice variants events between CA and AA, we selected a representative group of genes for qRT-PCR cross validation analysis. For normal *vs*. HCV+ (cirrhotic state), we selected the following genes: *SAA1*, *AOX1* and *SLC13A5*. Representative examples of the amplicon binding sites for the PCR primer sequences are shown in Supplementary Figures 1 and 2. For HCCN *vs*. HCV+HCC (tumor stage), the following genes were selected: *PCNA-AS1*, *IFI30*, *DBA2*, *ROBO1*, and *SNORD82*. The expression of these eight genes was validated by qRT-PCR using an independent test set of 24 liver and tumor tissue samples (12 CA and 12 AA). The qRT-PCR results are shown in Tables 5A and 5B. The data suggest that good concordance of the results is seen using HTA2.0 arrays and qRT-PCR analysis. However, there is a distinct difference in *SAA1* expression level between CA & AA samples (Table 5A). The overall fold change (FC) of *SAA1* in CA samples has a positive value because the overall gene expression in HCV+ cirrhotic liver is down compared to normal (Table 1A) resulting in a positive fold-change (FC) value. Although the overall FC (qRT-PCR) in AA samples (Table 5A) has a positive value, it is actually lower than CA, because the overall gene expression in HCV+ cirrhotic liver is higher in CA, thus lower value of FC is seen. Similar profile is seen in genes expressed in HCCN *vs*. HCV+HCC (tumor state): *PCNA-AS1*, *ROBO1*, *DAB2*, and *IFI30* (Table 5A, lower part). As shown in Table 5B, *SAA1* has an overall SI positive value in both HTA2.0 and qRT-PCR analyses. However, the SI value in AA samples (qRT-PCR) is lower compared to CA. This relates to the overall gene signal being higher in HCV+ cirrhotic liver (Table 5A, upper), thus more sliced out (higher signal) compared to normal. These data suggest that the observed disparity in HCV-induced HCC seen in CA and AA tissue samples could be due, in part, to transcriptome diversity of specific genes like *SAA1, PCNA-AS1*, *IFI30*, *DBA2*, and *ROBO1*.

**Table 5A:** qRT-PCR validation of 8 selected DEGs

Disease Stage	Gene Symbol	Accession Number	Fold Change (FC)			
			HTA 2.0		qRT-PCR	
Normal vs. HCV+			CA	AA	CA	AA
	*SAA1*	NM_000331	3.36	NA	3.12	2.0*
	*AOX1*	NM_001159	3.45	NA	3.10	3.3
	*SLC13A5*	NM_001143838	3.27	NA	3.51	3.0
HCCN vs. HCV+HCC	*PCNA-AS1*	NR_028370	3.53	NA	3.2	0.99*
	*ROBO1*	NM_001145845	2.20	NA	2.9	0.20*
	*DAB2*	NM_001244871	2.20	NA	3.0	0.55*
	*IFI30*	NM_001244871	2.21	NA	2.0	0.72*
	*SNORD82*	NR_004398	-2.20	NA	-2.0	-2.0

CA: Caucasian American; AA: African American.

*p<0.05; mean average of 3 biological replicates from each cohort.

**Table 5B:** qRT-PCR validation of alternative splicing of 3 selected genes

Disease Stage	Gene Symbol	Accession Number	Splicing Index (SI)			
			HTA 2.0		qRT-PCR	
Normal vs. HCV+			CA	AA	CA	AA
	*SAA1*	NM_000331	10.77	NA	9.12	3.21*
	*AOX1*	NM_001159	-2.55	NA	-2.10	-1.38
	*SLC13A5*	NM_001143838	-1.37	NA	-1.61	-1.12

CA: Caucasian American; AA: African American.

*p<0.05; mean average of 3 biological replicates from each cohort.

### Hepatocyte nuclear factor 4α (HNF4α) and serum amyloid A1 (SAA1)-associated protein staining patterns in liver and tumor tissue samples

**Figure 2:**
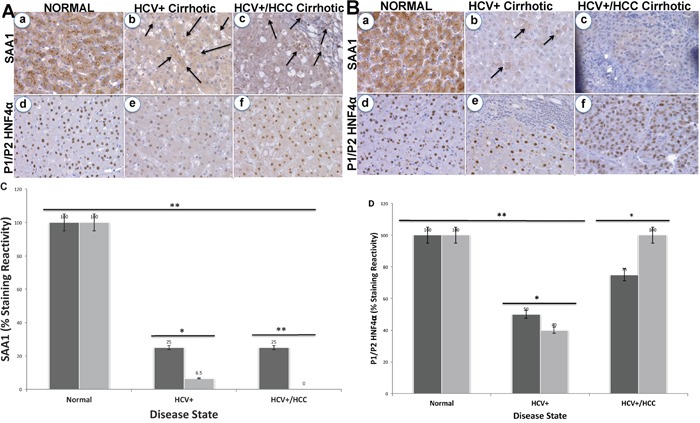
Immunohistochemical staining of SAA1 and P1/P2-HNF4α **(A)** Normal (a and d, respectively), HCV+ cirrhotic (b and e, respectively), and HCV+/HCC cirrhotic (c and f, respectively) in CA. **(B)** Normal (a and d, respectively), HCV+ cirrhotic (b and e, respectively), and HCV+/HCC cirrhotic (c and f, respectively) in AA. Bar graphs = % staining reactivity (Y-axis) *vs*. disease state (X-axis) for SAA1 **(C)** and P1/P2-HNF4α **(D)**. Black bar = CA; Gray bar = AA (*n*=3 – 4 tissue sections from 24 paraffin embedded tissue blocks ± S.E; *p<0.05; **p<0.001).

**Figure 3:**
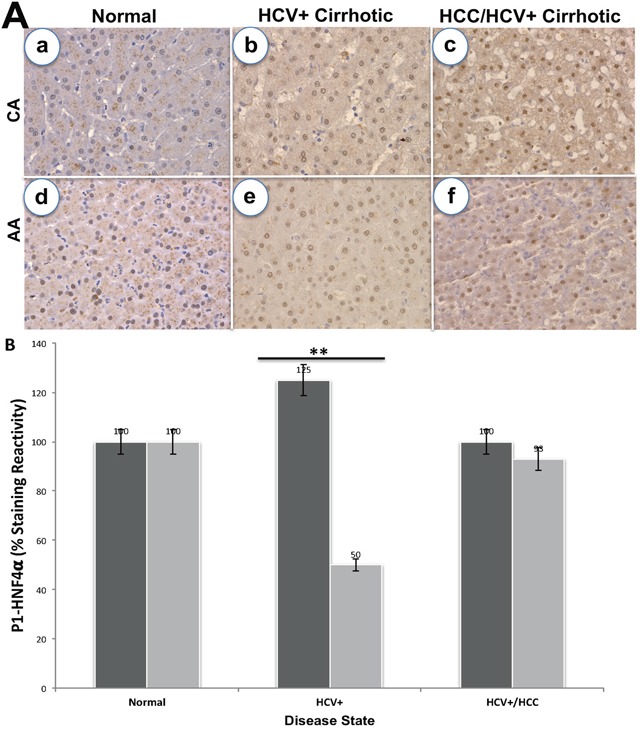
Immunohistochemical staining of P1-HNF4α **(A)** Staining in normal, HCV+ and HCC for CA (a-c) and AA (d-f) tissue samples. **(B)** Bar graphs = % staining reactivity (Y-axis) *vs*. disease state (X-axis) for CA, black bar and AA, grey bar (*n*=3 – 4 tissue sections from 24 paraffin embedded tissue blocks ± S.E; *p<0.05; **p<0.001).

Since *SAA1* is transcriptionally regulated by *HNF4α* [26], we examined the staining patterns of both proteins in 72 tissues sections for CA and AA using immunohistochemical analysis (Figures 2 and 3). Intense staining for SAA1 and P1/P2-HNF4α was observed in normal liver tissues for both CA (Figure 2Aa, and 2Ad) and AA (2Ba, and 2Bd). In contrast, the staining reactivity for both proteins showed a tendency to decrease in HCV+ cirrhotic livers of AA (Figure 2Bb, and 2Be) compared to CA (2Ab, and 2Ae). As shown in Figure 2C and 2D, the percentage of reactivity for SAA1 and P1/P2-HNF4α are 6.5 and 40 in AA, whereas in CA they are 25 and 50, respectively. Likewise, the staining patterns for both SAA1 and P1/P2-HNF4α in HCC are different in AA compared to CA samples. In AA tumor samples, there was no staining detected for SAA1 (Figure 2Bc), whereas intense staining was detected for P1/P2-HNF4α (Figure 2Bf). For CA tumor samples, staining was detected for both proteins, although less than what is detected in normal tissues (Figure 2Ac, and 2Af). Figure 3A illustrates the staining pattern of P1-HNF4α in tissue samples for both CA and AA. In HCV+ tissues, the percentage reactivity of P1-HNF4α is higher in CA (125%), and lower in AA (50%). There is no clear difference in HCC staining reactivity of P1-HNF4α between CA and AA.

## DISCUSSION

We previously showed [12] that there are distinct alterations in the expression of transcripts and proteins exist in CA liver and tumor tissue samples based on HCV disease state. However, the levels of expression were different when the results were cross- validated on tissue samples of AA cohort. The aim of the current study was to follow up on these findings and investigate, at the whole transcriptome level, the extent to which splice variant events may play a role in this genomic diversity of HCV disease state and racial disparity. Alternative splicing of mRNA is a major mechanism that generates diverse mRNA transcript isoforms from a single gene, and subsequently differentiates proteins to have varying cellular processes [19–23]. These variants are targeted as biomarkers in disease diagnosis, prognosis and treatment [27–29].

In the present study, genome-wide analyses of genes and alternative splicing events of human liver and tumor tissues were performed using the newly developed Affymetrix Human Transcriptome 2.0 arrays (HTA 2.0). With a high density of oligonucleotide probes, these arrays cover the exonic regions of human genome as well as junction regions between adjacent exons. Many changes were apparent in HCV+ cirrhotic *vs*. normal livers, even more so than HCV+HCC *vs*. HCCN. This may indicate that HCV+ cirrhotic livers, as a type of intermediary lesion in HCV disease progression, already exhibited strong signs of alternations. From the molecular changes evidenced in HCV+ (Figure 1A), it is clear that HCV+ cirrhotic livers are not merely accumulating alterations that will be found in HCV+HCC (Figure 1B). Possibly, the evolution to HCC follows a more strictly clonal expansion, which may select for gene changes important for clonal growth while eliminating less relevant modifications. According to this hypothesis, HCV+ cirrhotic livers may have different outcomes, some evolving toward cancer (HCC), whereas others could be prone to disappearance. In this case, we were able to identify more genes expressed in normal *vs*. HCV+ (636 DEGs), whereas only 61 DEGs were detected in HCCN *vs*. HCV+HCC. No overlap of genes was detected between the two disease states.

Tables 1A & 1B show specific gene expression alterations in normal *vs*. HCV+. The signature of 350 probes corresponding to downregulated genes in HCV+ compared to normal is shown in Table 1A. Among the highest down- regulated genes are: *AVR1A*, *SAA2*, *MT1F*, *CFHR5*, *SLITRK3*, *CLEC4M*, *SAA1*, *CPN1*, *TIMD4*, *GPR125*, and *AOX1*. Most of these genes have not been described to be associated with HCV+ cirrhotic livers, although several of the changes agreed to previous reports including variations in the expression levels of *SAA1*, *SAA2* or *MT1F* [30–33]. For example, *SAA1* and *SAA2* are well-known acute phase reactants, and their serum levels were shown to be down regulated in HBV-associated HCC patients compared to healthy individuals [34]. In our study, both *SAA1* and *SAA2* are down regulated in HCV+ liver compared to normal (Figure 1A). As tumor suppressor, metallothionein 1F (*MT1F*) has been shown to be down regulated in several tumors as part of cancer initiation and/or progression [35]. The signature of 286 probes corresponding to upregulated genes in HCV+ compared to normal is shown in Table 1B. Among the highest upregulated genes are: *AKR1B10*, *IFI27*, *IL8*, *VTRNA1-1*, *SPP1*, *GDF15*, *CXCL10*, *IGLC7*, and *LGALS4*. The expression of these genes is known to be strongly associated with HCV-induced liver cirrhosis and/or HCC [36–45]. In Figure 1A, both *SPP1* and *IL8* are upregulated in HCV+ cirrhotic liver compared to normal.

The signature of 61 probes corresponding to genes showing expression alterations in HCCN *vs*. HCV+HCC is shown in Table 2. In this disease state, 47 genes (77%) are upregulated, whereas 14 genes (23%) are downregulated. Among the top deregulated probes, *PCNA-AS1* has been found to be the most up-regulated probes in HCV+HCC compared to HCCN, whereas *SNORD82*, among the downregulated probes (Figure 1B). Both genes are considered long non-coding RNAs (lncRNAs) and well recognized to play major regulatory roles in disease development. For example, *PCNA-AS1* was shown to act as an upstream regulator in HCC [46], and *SNORD82* has been found to be involved in the development of prostate and breast cancers [47, 48]. Ingenuity Pathway Analysis (IPA) was performed using Ingenuity software, as we reported previously [12] to understand the correlation between the canonical biological pathways and the deregulated genes identified in this study. Among the top 5 canonical pathways for normal *vs*. HCV+ state (Table 5A) was *Hepatic Fibrosis/Satellite Cell Activation* (*p*=4.25E-04). In hepatic fibrosis, hepatotoxins like HCV initiate a cascade of stress related pro-inflammatory events, which eventually activate Hepatic Stellate cells (HSCs). Activated HSCs secrete cytokines that perpetuate their activated state. Continued liver injury results in an accumulation of activated HSCs, which in turn synthesize large amount of extracellular matrix (ECM) proteins, leading to severe fibrosis and eventually liver cirrhosis. *SAA1* and *SAA2* genes are among the molecules activated in this disease state (acute phase reactants), and both are down regulated indicating a possible involvement in disease initiation to HCC. For HCCN *vs*. HCV+HCC state (Table 5B), *GADD45 Signaling* was the top pathway identified (*p*=2.93E-06). It has been implicated in stress signaling response that can result in cell cycle arrest, DNA repair, cell survival, senescence, and apoptosis. This response is mediated via a complex binding to several proteins involved in these processes, including PCNA and thus PCNA-ASI was found to be upregulated in HCC (Figure 1B).

We next validated the expression of 8 DEGs by real-time qRT-PCR using independent samples for CA and AA, as shown in Table 5A. Although it is clearly shown in this table that there is good concordance in results obtained using both platforms, the level of *SAA1* in AA samples (normal *vs*. HCV+ state) is significantly lower than that of CA (*p*<0.05). Thus, immune response to chronic HCV infection may play a crucial role in HCV racial disparities. Four (*PCNA-AS1*, *ROBO1*, *DAB2* and *IFI30*) out 5 transcripts with increased expression in HCCN *vs*. HCV+HCC state (Table 2) were found to be significantly lower (*p*<0.05) in AA compared to CA samples. Thus, in addition to the immune response-associated genes, these genes could also play a role in HCV/HCC racial disparities seen between CA and AA samples, and might be valuable markers for early diagnosis of the disease based on racial background of patients.

Since *SAA1* (acute response reactant) is transcriptionally regulated by *HNF4α* [49] we validated the expression of both using immunohistochemical analysis. *HNF4α* is a member of the superfamily of ligand-dependent transcription factors (TFs) and master regulator of tissue-specific gene expression in the liver [50]. It inhibits progression of HCC in mice [17, 18]. There are two alternative promoters that drive expression of *HNF4α* gene (P1 and P2) and give rise to HNF4α isoforms that differ by 16-38 amino acids in their terminal region [51]. While the different isoforms have identical DNA and ligand binding domains, there subtle yet significant functional differences between the HNF4α isoforms. Both P1- and P2-driven *HNF4α* are expressed in the fetal liver but only P1- *HNF4α* is expressed in the normal adult liver [52], and P1- *HNF4α* is down regulated in human HCC while P2- *HNF4α* is upregulated [51]. Furthermore, P1- *HNF4α* is known to repress the activation of the P2 promoter [51], which could explain the switch between the two isoforms. In this study, we used both H1415 and K9218 monoclonal antibodies to detect P1/P2- and P1-promoter-driven *HNF4α*, respectively, in the liver and tumor samples to determine how the expression of these two isoforms may play a role in SAA1 expression patterns. Our data in Figure 2 clearly indicate that staining reactivity of SAA1 and P1/P2-HNF4α is altered based on HCV disease state and race. For example, staining reactivity (%) for SAA1 (Figure 2C) in CA is 25% for both HCV+ cirrhotic and HCC states, whereas in AA samples it is only 6.5% and 0.0%, respectively. This indicate that the marker for “acute inflammatory phase” is much lower in HCV+ of AA compared to CA cohort. As shown in Figure 2D, the staining reactivity of P1/P2- *HNF4α*, which is a measure of both isoforms, is lower in HCV+ for both CA and AA tissue samples. However, it is clearly shown in Figure 3B that the low staining reactivity is related to P1- *HNF4α* isoform, and mainly in AA tissue samples. These data clearly indicate that the acute inflammatory phase as measured by SAA1 level is severely compromised in AA compared to CA as a result of dysregulation of *HNF4α* isoforms. Our results also show that changes in splicing profiles in normal *vs*. HCV+ state could possibly contribute to the observed HCV disease state racial disparity (Table 3). The alternative splicing events of three genes (*SAA1*, *AOX1* and *SLC13A5*) from the 28-gene set (Table 3) were confirmed by real-time qRT-PCR in normal *vs*. HCV+ state. Specifically, we validated the expression of *SAA1*, *AOX1*, and *SLC13A5*. For *SAA1*, the expression of exon 1 to 2 and exon 1 to 3 (Supplementary Figure 1), for *AOX1* 4 to 5, and the exon 12 to 13, for *SLC13A5* exon 10 to 12 (Supplementary Figure 2). We found that the splicing index (SI) of *SAA1* is significantly lower (*p*<0.05) in AA compared to CA (Table 5B). This suggests that splicing events occurred mainly in specific disease state (HCV+ cirrhotic) predominantly in AA cohort. The role played by these alternative splice products in HCV+ will thus require further investigations, together with the other alternative transcripts detected. In sum, our study suggests that altered gene expression, and splice variants are important events in HCV racial disparities between Caucasian and African Americans.

In conclusion, our genomic variants study showed that genes were differentially expressed between HCCN and HCV+HCC but, also, to a large extent, between normal and HCV+ (cirrhotic) state. Many of these genes are involved in biological pathways pertinent to the overall pathophysiological response to HCV infection. The observation that several splice variants were deregulated in normal *vs*. HCV+ is certainly in line with the recent observations showing that the pre-mRNA splicing machinery may be profoundly remodeled during HCV disease progression, and may, therefore, play a major role in the disease outcome. Target validation analyses showed that some of these genes are significantly deregulated especially in AA compared to CA tissue samples. These observations suggest that socioeconomic factors may not fully explain the differences in HCV racial disparity, but rather biological/genetic factors should also be considered. Further analyses will be required to determine if these gene variants are predictive markers of the pathophysiological evolution in HCV disease progression. It would be of great interest to determine whether our differentially expressed genes and splice variants are under some kind of coordinated control. This certainly will allow for the development of next generation therapeutic care management for HCV disease state based on racial/ethnic backgrounds of patients.

## MATERIALS AND METHODS

### Sample preparation and data analysis

Total RNA was extracted from 12 tissue samples of Caucasian individuals (3 normal livers, 3 HCV+/HCC- (cirrhotic livers), 3 HCV+/HCC+ (cirrhotic tumors) and 3 normal adjacent tissue matched pairs HCCN) using the RNeasy mini kit (Qiagen, Valencia, CA, USA) and quantified using Nanodrop ND-100 Spectrophotometer (Thermo Fisher Scientific, Waltham, MA, USA), as previously reported [12]. RNA samples were then subjected to RNA amplification using the SensationPlus FFPE Amplification and WT Labeling Kit (Affymetrix Inc., Santa Clara, CA, USA), as previously reported [53, 54]. The biotin double-stranded cDNA products were hybridized to Affymetrix HTA 2.0 arrays using an Affymetrix hybridization kit. Hybridized HTA 2.0 arrays were scanned with an Affymetrix GeneChip® 3000 fluorescent scanner. Image generation and feature extraction was performed using Affymetrix GeneChip Command Console Software. The raw data (.*CEL) were analyzed using the Transcriptome Analysis Console (TAC) 2.0 software, which allows for the identification of differentially expressed genes (DEG) & exons and the visualization of alternative splicing events for determining possible transcript isoforms that may exist in samples.

For microarray data analysis, two parallel analyses (gene-level and alternative splicing level) were performed. Data were normalized using quantile normalization, and background noise was detected using Detection Above Background (DABG) algorithm. Only the probesets characterized by a DABG *p*-value <0.05 in at least 50% of the samples were considered for statistical analysis. We performed an unpaired Student's *t*-test to compare gene intensities between normal vs. HCV+ and HCCN vs. HCV+HCC. Genes were considered significantly regulated when Fold Change (FC), linear <-2.0 or >+2.0 and ANOVA *p*-value (condition pair) <0.05. Analysis of the splicing level was also performed using TAC 2.0 software, which determines among other parameters, the Splicing Index (SI) of a gene. The SI corresponds to a comparison of gene-normalized exon-intensity values between the two analyzed experimental conditions [55]. Additional criteria used beside SI: *q*-value <0.05, a gene is expressed in both conditions (normal vs. HCV+, and HCCN vs. HCV+HCC), a Probset Ratio (PSR)/Junction must be expressed in at least one condition, and a gene must contain at least one PSR value.

### Reverse transcription PCR validation

Validation of 8 selected differentially expressed genes (DEGs) and splice variants was performed on 24 independent tissue samples (12 CA, and 12 AA) at various disease state (normal, HCV+ and HCC). mRNA levels were measured using the SYBR-GREEN quantitative RT-PCR (qRT-PCR) method as previously reported [12] by the ABI 7900HT Fast Real Time PCR System (Applied Biosystems). cDNAs were amplified using specific primers indicated in Supplementary Table 1; data results were normalized against alpha-ACTIN (ACTIN1), beta-2-Microglobin (B2M), and glyceraldehyde 3-phosphate dehydrogenase (GAPDH). Relative RNA levels of genes were calculated using the comparative Ct method 2^-ΔΔCt^ [56]. For splice variants, alt-spliced (A) and constitutive (C) exons were identified in TAC 2.0, and qRT-PCR primer sets were designed using Primer3 (http://www.ncbi.nlm.nih.gov/tools/primer-blast/) as shown in Supplementary Table 1. By designing specific primer pairs for constitutively expressed flanking exons (Supplementary Figure 1 and 2), it is possible to simultaneously amplify isoforms that include or skip the target exon [57]. The identities of variant specific amplicons were simultaneously verified and quantitated by melt curve analysis, and the products were confirmed either present or absent using agarose gel electrophoresis. Splice Index (SI) was calculated for (A) by normalizing fold change (FC) to the average FC of (C) for each splicing event. For amplicon spanning exons 4-5 in *AOX1* (Supplementary Table 1), the calculated FC (A)/average FC (C) value is less than 1 (0.47), indicating decreased exon 5 inclusion in Normal vs. HCV+. This is finally reported as -1/0.47 = -2.1, as a negative number (Table 5B). For *SAA1*, the reported positive SI number (9.12) indicates increased exon 3 inclusion in Normal vs. HCV+. Each sample was measured in triplicate and values were reported as average.

### Immunohistochemistry

Study tissue blocks (24 samples, including 3 normal; 3 HCV+, 3 HCCN and 3 HCV+/HCC for CA and AA, respectively) were selected after histopathologic review by pathologists. Three 4-tissue sections were selected from each block (total = 96 tissue slides). All of the tissue slides were treated to heat induced epitope retrieval (HIER) in a decloaker (BIocare Inc.) using HIER-L solution (citrate buffer, pH 6.0, Thermo Fisher). Detection for serum amyloid A1 protein (SAA1) and hepatocyte nuclear factor 4-alpha (HNF4α) isoforms was performed by incubating slides in a rabbit anti-mouse antibody (SAA1, Clone # 902738, R&D Systems, Cat # MBA30191, dilutions 1:50), (P1/P2-HNF4α, Clone # H1415, R&D Systems, Cat # PP-H1415-00, dilutions 1:100) or (P1-HNF4α, Clone # K9219, Cat # PP-K9218-00, dilutions 1:100) overnight at 4°C followed by incubation in a horseradish peroxide-conjugated anti-rabbit antibody, then developing with 3,3’-diaminobenzidine tetrahydrochloride chromogen. For negative control, the primary antibodies were replaced with PBS. Liver sections were used as positive controls. Staining reactivity for each protein/tissue slide was graded by two pathologists (MMY and SB) as consensus using a semi-quantitative scoring system (0 – 4) as previously reported [58]. The staining reactivity of 3-4 tissue slides was plotted for SAA1, P1/P2- and P1- HNF4α.

### Pathways, functional enrichment and interactive network analysis

Gene networks and canonical pathways representing key genes were identified through the use of QIAGEN'S Ingenuity Pathway Analysis software (IPA, QIAGEN Redwood City, www.qiagen.com/ingenuity, content version 18841524, release date 06/26/2014) as previously reported [12]. Briefly, the data sets containing gene identifiers and corresponding fold change and *p*-values were uploaded into the web-delivered application and each gene identifier was mapped to its corresponding gene object in the IPA software. Fisher's exact test was performed to calculate a *P*-value assigning probability of enrichment to each biological function and canonical pathway within the IPA library.

### Statistical analysis

The data were expressed as mean±SE, and analyzed with the Student's *t*-test between two groups. Changes were considered statistically significant if the *P*-value was <0.05.

### Ethics statement

Washington State University (WSU) Office of Research Assurances has found that the study is exempt from the need for the Institutional Research Board (IRB) approval. Thirty-six snapped frozen tissue samples (12 included in the original analysis and 24 for target validation study), as well as 25 tissue sections from formalin-fixed paraffin-embedded blocks were obtained from the IRB approved University of Kansas Medical Center Liver Center Tissue Bank. All specimens with anonymized identifiers were histopathologically confirmed by a pathologist.

## SUPPLEMENTARY MATERIALS FIGURES AND TABLES



## Abbreviations

HCV: Hepatitis C virus
HCC: Hepatocellular carcinoma
HTA2.0: Human Transcriptome Array 2.0
HCV+: HCV positive cirrhotic liver
HCV+HCC: HCV positive liver tumor
HCCN: Tumor adjacent normal tissue
CA: Caucasian American
AA: African American
AS: Alternative splicing
DEGs: Differentially expressed genes
IPA: Ingenuity pathway analysis
qRT-PCR: Quantitative real-time-PCR
FC: Fold Change
SI: Splicing Index
PSR: Probeset Ratio
IHC: immunohistochemistry
