# Supplementary material for: Genomic variants link to hepatitis C racial disparities
Source: Oncotarget. 2017 Aug 1;8(35):59455–75. doi: 10.18632/oncotarget.19755 (PMC5601746; doi:10.18632/oncotarget.19755)
Supplement: Supplementary file 1 [file oncotarget-08-59455-s001.pdf]

Genomic variants link to hepatitis C racial disparities

SUPPLEMENTARY MATERIALS

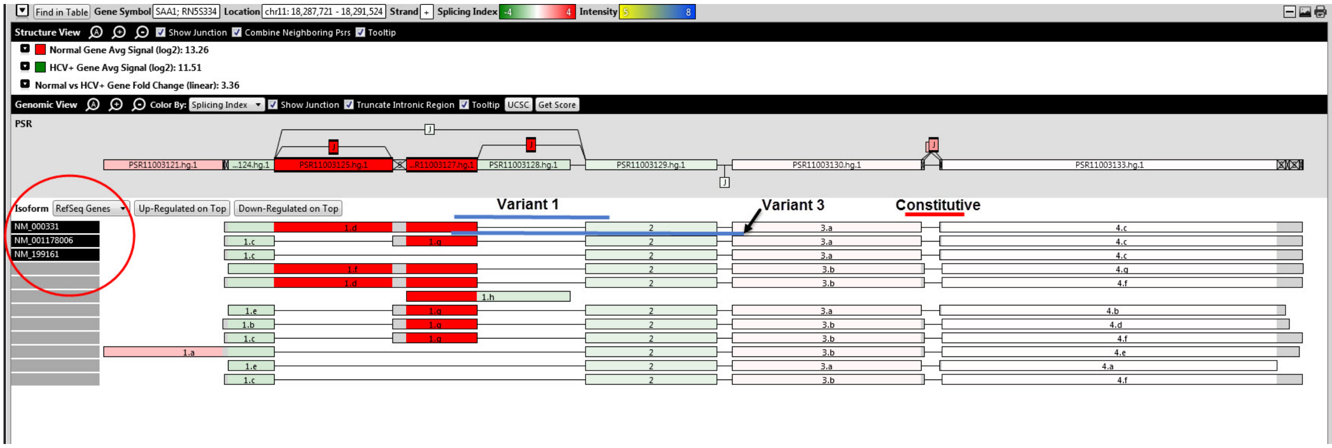

Supplementary Figure 1: Genomic view of primer design for *SAA1* alternative splicing qRT-PCR validation. Gene structure view of *SAA1* from TAC 2.0 software (to scale) and approximate positions of qRT-PCR amplicons (not to scale) are shown. The positions of primer sets in Supplementary Table 1 (constitutive C and alt-spliced A) are shown. Splicing Index (SI) from qPCR data is calculated for alt-spliced amplicons by normalizing their fold change to the average fold change of constitutive amplicons. Splice Indices = 9.12 and 3.21 for CA and AA, respectively. In effect, SI is a fold-change measure of the alt-spliced event.

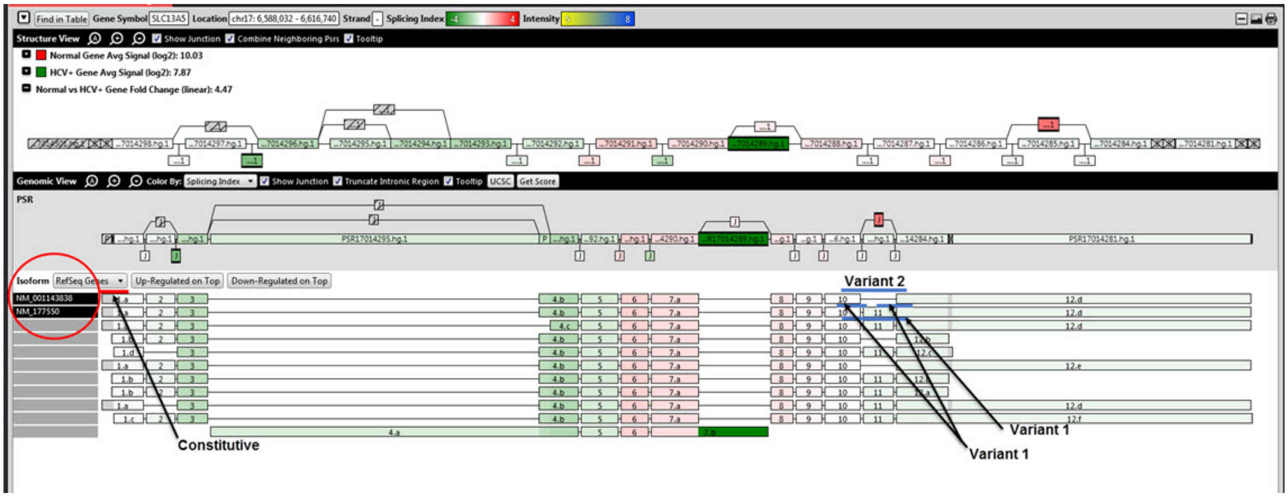

Supplementary Figure 2: Genomic view of primer design for *SLC13A5* alternative splicing qRT-PCR validation. Gene structure view of *SLC13A5* from TAC 2.0 software (to scale) and approximate positions of qRT-PCR amplicons (not to scale) are shown. Splicing Index (SI) from qPCR data is calculated for alt-spliced amplicons by normalizing their fold change to the average fold change of constitutive amplicons. Splice Indices = -1.61 and -1.12 for CA and AA, respectively.

Supplementary Table 1:PCR primer sequences

Name*	Amplicon binding site	Exon	Forward (5'-3')	Reverse (5'-3')	Annealing temp. (°C)
SAA1cons	409 to 640	3 to 4	AGTGATCAGCG ATGCCAGAG	CCATTGTGTA CCCTCTCCCC	60
SAA1v1as	29 to 257	1	CAGATCAGGT GAGGAGCACAC	ACGAAAAGAA GCTTCGGCTG	60
SAA1v3as	19 to 118	1 to 3	GCTACAGCACAGA TCAGTTATCC	CCCGTGAGAA GCTTCATGGT	60
AOX1cons	3042 to 3145	26	ATGGCCATGTCTT CCTACTCC	GGAAACTTCA GGGGGACCAT	60
AOX1ex4.5as	309 to 427	4 to 5	CCAGCCAATG CCTGTCTGAT	CACTTGGCAAT CCTCTCCTGAA	60
AOX1ex12.13as	1167 to 1236	12 to 13	GGGGGACACAT CATTAGCAGG	GGGTACAGTTA CCCACAGCC	60
SLC13A5cons	14 to 119	1	TGTGGCCCCTT CTTAAGCCC	ACTTGGAGACA TAGCTCAGCG	60
SLC13A5v1as	1518 to 1645	10 to 11	CCTCCATGTC TCGCTCCATC	GTGCCCATAG GTGAACACGA	60
SLC13A5v2as	1423 to 1536	10 to 11	GGCAGCCATCA CCTTGATCT	CCTGTTTTCA CCATGGAGGC	60
SNORD82cons	4 to 75	1	GCACAAATGATGAA TAACAAAGGGA	ACAGCACATCA GCACACTACA	60
PCNA-AS1cons	266 to 357	1	TAGCTGGTTTCG GCTTCAGG	CGTAGCAGAG TGGTCGTTGT	60
IFI30cons	363 to 457	2 to 4	TACGGAAACG CACAGGAACA	CAGGCCTCCA CCTTGTTGAA	60
DBA2cons	418 to 519	1 to 2	CAATAGCTAGCC GGTGTCTGT	GGCAGCAAAC CTCAGTACCA	60
ROBO1cons	112 to 304	1 to 3	TCTGCACTAC GGAGCCTCT	TTCAGGGTCTGG AATAAGCTGG	60
**ACTINalpha**	**1652 to 1768**	**12 to 13**	**CAGGACCGTGT GGAGCAGATTG**	**CAGATTGTCCC ACTGGTCACAG**	**60**
**B2M**	**348 to 426**	**2 to 3**	**TGAGTATGCCT GCCGTGTGAAC**	**TGCTGCTTACAT GTCTCGATCCC**	**60**
**GAPDH**	**1091 to 1239**	**8 to 9**	**TGCCCTCAAC GACCACTTTG**	**CTCTTCCTCTTG TGCTCTTGCTG**	**60**

Bold = Reference genes.
cons = constitive.
v1-v3 = variant 1-3.
as = alternative splicing.

Supplementary Table 2: Clinical characteristics of the study cohort

Variables	Caucasian American (CA)			African American (AA)		
	Normal (HCV-)	HCV+ (CIR)	HCV+/HCC	Normal (HCV-)	HCV+ (CIR)	HCV+/HCC
Age, years, mean (range)	48.5 (38-64)	51 (32-61)	55.3 (48-67)	42.3 (38-50)	48.3 (33-62)	59.3 (56-66)
Male sex, n (%)	4 (67)	4 (57)	5 (83)	3 (100)	3 (67)	3 (67)
Laboratory values, mean (SD)						
ALBUMIN(g/dL)	NA	3.1(0.7)	2.77(0.3)	NA	2.6(0.5)	1.6(0.6)
AST(U/L)	NA	47(14.4)	91(104)	NA	75.6(94)	88(10)
ALT(U/L)	NA	27(1.5)	64(86.3)	NA	51(5.4)	66 (4.6)
BIL-T(mg/dL)	NA	4(1.7)	2.1(1.1)	NA	5.3(3.1)	2.2(0.8)
HEMOGLOBIN	NA	9.1(1.8)	13.9(1.7)	NA	9.3(2.1)	12(1.8)

CIR= Cirrhotic; HCV+/HCC = HCV-associated Hepatocellular carcinoma.
HCV genotype =Ia.
